# Role of “Second Look” Lymph Node Search in Harvesting Optimal Number of Lymph Nodes for Staging of Colorectal Carcinoma

**DOI:** 10.1155/2018/1985031

**Published:** 2018-04-02

**Authors:** Ameer Hamza, Ramen Sakhi, Sidrah Khawar, Ahmed Alrajjal, Jacob Edens, Muhammad Siddique Khurram, Uqba Khan, Susanna Szpunar, Paul Mazzara

**Affiliations:** ^1^St. John Hospital and Medical Center, Detroit, MI, USA; ^2^Vanderbilt University Medical Center, Nashville, TN, USA

## Abstract

As with other malignancies, lymph node metastasis is an important staging element and prognostic factor in colorectal carcinomas. The number of involved lymph nodes is directly related to decreased 5-year overall survival for all pT stages according to United States Surveillance, Epidemiology, and End Results (SEER) cancer registry database. The National Quality Forum specifies that the presence of at least 12 lymph nodes in a surgical resection is one of the key quality measures for the evaluation of colorectal cancer. Therefore, the harvesting of a minimum of twelve lymph nodes is the most widely accepted standard for evaluating colorectal cancer. Since this is an accepted quality standard, a second attempt at lymph node dissection in the gross specimen is often performed when the initial lymph node count is less than 12, incurring a delay in reporting and additional expense. However, this is an arbitrary number and not based on any hard scientific evidence. We decided to investigate whether the additional effort and expense of submitting additional lymph nodes had any effect on pathologic lymph node staging (pN). We identified a total of 99 colectomies for colorectal cancer in which the prosector subsequently submitted additional lymph nodes following initial review. The mean lymph node count increased from 8.3 ± 7.5 on initial search to 14.6 ± 8.0 following submission of additional sections. The number of cases meeting the target of 12 lymph nodes increased from 14 to 69. Examination of the additional lymph nodes resulted in pathologic upstaging (pN) of five cases. Gross reexamination and submission of additional lymph nodes may provide more accurate staging in a limited number of cases. Whether exhaustive submission of mesenteric fat or fat-clearing methods is justified will need to be further investigated.

## 1. Introduction

Lymph node metastasis is one of the most important prognostic factors in colorectal cancer [1, 2]. It is not only a necessary element of TNM staging but is also the most important factor in determining appropriate therapy [3, 4]. The likelihood of finding any lymph node metastases increases with increased lymph node count, but at some level yields diminishing returns. The optimal number of lymph nodes providing accurate staging prior to reaching the point of diminishing returns has been a subject of much debate. Harvesting of 12 lymph nodes is now considered the minimum target for accurate staging of colectomy specimens in patients with colorectal cancer [1]. We wanted to study whether additional efforts to reach this target had any effect on pathologic staging.

## 2. Materials and Method

Following approval by the institutional review board (IRB), we ran a retrospective search of the computer database for colorectal specimens received in the Department of Pathology at St. John Hospital and Medical Center in Detroit, MI. Our study group consisted of colorectal carcinoma resection specimens obtained during the period 2012–2016 in which additional sections were submitted in an attempt to retrieve additional lymph nodes. Of the 323 colorectal specimens in which additional sections were submitted, 103 were for the purpose of examining additional lymph nodes. Four of the cases were excluded from the study because the tumor was an unexpected finding, and therefore, neither the surgery nor the initial gross handling of the specimen was performed with staging as a primary objective. Data such as pathologic TNM staging and number of lymph nodes examined prior to and after submission of additional lymph nodes were compared, and any potential effect on treatment was determined. The data were analyzed using Student's *t*-test and the Mann–Whitney test. All data were analyzed using SPSS versus 24.0, and a *p* value of 0.05 or less was considered statistically significant.

## 3. Results

A total of 99 large bowel specimens resected for colorectal carcinoma were identified in which additional lymph nodes were submitted following a second detailed search of the gross specimen. The mean number of lymph nodes found initially in these specimens was 8.3 ± 7.5. The mean number of lymph nodes increased to 14.6 ± 8.0 (a mean increase of 6.3 ± 4.7) with the submission of additional lymph nodes following a repeat search. Of the 99 colons in the study, the target number of twelve or more lymph nodes was initially achieved in only 14 cases. Following a second look and submission of additional lymph nodes, the target number was attained in 69 cases. Less than 12 lymph nodes were harvested in 30 cases despite a second attempt at lymph node procurement. A mean of 7.9 ± 5.9 cassettes of lymph nodes was initially submitted (1.3 ± 0.8 lymph nodes per cassette) compared to a mean of 12.7 ± 13.4 additional cassettes, yielding 0.79 ± 0.78 lymph nodes per cassette. When comparing the number of lymph nodes retrieved in patients with and without neoadjuvant therapy, the number of lymph nodes was less in those who received neoadjuvant therapy (7.8 ± 5.1 versus 8.7 ± 8.7 initially and 14.0 ± 6.3 versus 15.0 ± 9.0 following a second look), but the differences were not statistically significant (Table 1). When we evaluated the role of the individuals performing the gross exam, residents retrieved higher initial and total lymph node counts compared to pathologist assistants (8.4 ± 8.0 versus 8.0 ± 6.0 initially and 15.1 ± 8.3 versus 13.0 ± 6.8 following second look), but again the differences were not statistically significant (Table 2). Submission and examination of additional lymph nodes resulted in the identification of additional lymph node metastases in 9 cases, but the final (pN) stage was upgraded in only 5 cases. However, in only 3 cases was the stage grouping upgraded (stages II to III). Data on these 9 cases are summarized in Tables 3 and 4. The three cases that were upstaged were among the 55 specimens in which a minimum of 12 lymph nodes were retrieved following the second search. The results of the 55 cases in which the target of 12 lymph nodes was reached after a second look are summarized in Table 5. No additional positive lymph nodes were identified in the 30 cases in which less than 12 lymph nodes were retrieved even after a second attempt.

## 4. Discussion

Lymph node status is an integral part of TNM staging [5, 6]. Increasing numbers of positive lymph nodes (higher N stage) progressively decrease the 5-year overall survival for all T stages according to United States Surveillance, Epidemiology, and End Results (SEER) cancer registry database. A multicenter observational study [7] by National Cancer database in the United States and many other studies [8–16] show a statistically significant reduced overall and disease-free survival with decreased number of lymph nodes harvested in colorectal cancers. Based on different studies, the optimal number of lymph nodes for accurate staging of colorectal carcinoma ranges from six to twenty-one [7–10, 15–22]; however, 12 lymph nodes is the minimum standard specified by the National Quality Forum, and the College of American Pathologists also suggests that 12 is the minimal acceptable harvest from a careful dissection of a colorectal specimen [1, 6, 23–25]. The number of lymph nodes harvested is dependent upon many factors including the age of the patient, the segment and length of bowel, treatment with chemoradiation, and the skill of the surgeon and individual performing the dissection. The mean number of lymph nodes decreases with age [11, 18, 26–28], from proximal to distal segments of bowel [2, 8, 10, 18, 29–31], and with the use of neoadjuvant therapy [32–40]. It increases with increasing T-stage [2, 16, 31, 41] and is also associated with microsatellite instability [42]. The number of lymph nodes harvested is greater with tumors exhibiting microsatellite instability as compared to those that are microsatellite stable owing to a more intense host immune response resulting in activated, enlarged lymph nodes that are easier to detect [42]. Although one or more of these factors can have a negative impact on the number of lymph nodes retrieved, there are no concessions given for the number of lymph nodes required to reach the minimum standard which has been arbitrarily set at 12. There are no clear guidelines or consensus on how much effort and time should go into achieving that target or whether it makes any difference in patient management; however, according to NCCN guidelines, T3 N0 (stage II) patients with high risk of recurrence should receive neoadjuvant therapy [43]. Among different high risk factors, examining less than 12 lymph nodes is considered one of the high risk factors for recurrence as per NCCN guidelines [43].

Additional tissue sections contributed to the final diagnosis in only 3.8% cases in a recent study [44]. In our study, the subsequent search and submission of additional lymph nodes changed the pN designation in 5 of the cases. However, the clinical stage based on stage grouping remained the same in all but 3 cases. Therefore, the added effort, time, and expense of submitting additional lymph nodes were unlikely to have any impact on patient management in all but three patients.

Neoadjuvant therapy can lead to tumor necrosis with fibrous replacement and lymph node atrophy, resulting in more difficult lymph node dissection of the gross specimen. Rullier et al. [33] reported that neoadjuvant therapy not only reduced mean number of total lymph nodes harvested (reduced from 17 to 13, *p* = 0.001) but also reduced mean number of positive lymph nodes (reduced from 2.3 to 1.2, *p* = 0.001). In our study, neoadjuvant therapy decreased the lymph node yield, but the difference was not statistically significant (Table 1). The likely reason for this is that only specimens requiring second lymph node search were included in the study and every effort was made during the second look to achieve the desired minimum number of lymph nodes irrespective of neoadjuvant therapy. As for positive lymph nodes, our results were different from what was observed by Rullier et al. [33]. We found that mean number of positive lymph nodes was higher in patients who received neoadjuvant therapy (0.9 ± 1.7) compared to those who did not receive neoadjuvant therapy (0.6 ± 1.5). However, the difference was not statistically significant (Mann–Whitney test; *p* = 0.12). When considering only the 55 cases in which the target of 12 lymph nodes was achieved following a second attempt, a mean of 1.47± 2.1 positive lymph nodes was found in patients who received neoadjuvant therapy versus 0.6 ± 1.6 positive lymph nodes in patients who did not receive neoadjuvant therapy. In this group, the difference was statistically significant (Mann–Whitney test; *p* = 0.02).

The presence of lymph node metastases defines TNM stage III, irrespective of pathologic T stage. Due to the number of factors affecting the number of lymph nodes harvested, the lymph node ratio has been suggested to be a better indicator of disease severity [45]. This is defined as the ratio of positive lymph nodes to the total number of lymph nodes retrieved. There is no consensus on a numerical value for lymph node ratio, but available data suggests worsening survival with higher lymph node ratios [26–28, 46–50]. In our study, we focused on the utility of a second look lymph node search and did not collect data on patient outcome. When considering all 99 cases, the initial lymph node ratio was 0.06 which decreased to 0.05 following the second attempt at lymph node retrieval. For the 55 cases in which a minimum of 12 lymph nodes was achieved following a second search, the lymph node ratio decreased from 0.09 to 0.06.

There is no debate that harvesting more lymph nodes increases the likelihood of finding positive lymph nodes and thus upstaging a patient from stage I or II to stage III. According to population statistics, correctly upstaging a patient from stage II to III would increase the survival in both groups [51]. This is called the Will Rogers phenomenon [51]. It may result in more patients getting adjuvant therapy; however, survival benefit of giving adjuvant therapy to this particular group of patients who would have not been considered for adjuvant therapy otherwise is not known.

From pathologic stand point and grossing perspective, it was noted that second look lymph node search increases the lymph node yield and proportion of cases in which the minimum target number of 12 lymph nodes is achieved. However, a second search for lymph nodes requires more tissue sections per lymph node retrieved than the initial search, probably due to the fact that the lymph nodes are smaller and harder to find and more fat devoid of lymph nodes is submitted in an attempt to reach the target number. This has financial implications for the laboratory. The cost of processing one additional cassette in our laboratory is estimated to be $4.11. This includes the cost of the reagents, cassettes, glass slides, coverslips, and labels as well as the estimated labor costs of preparing the slides. This is not inconsequential since the mean number of additional sections is 12.7 which equates to an average additional cost of $52.20 for each specimen.

## 5. Conclusion

Accurate pathologic staging is critical in determining appropriate therapy and prognosis in colorectal cancer. The presence of lymph node metastases upstages pTNM stages I and II tumors into stage III. Therefore, identifying any lymph node metastases that may be present is vital. Examining a minimum of 12 lymph nodes has been established by the CAP and National Quality Forum for adequate staging. However, there are numerous factors that affect the number of lymph nodes retrieved, and this minimum target is not always reached. The amount of effort and expense that should be exercised to try to reach the target is unclear. We found that a second attempt at lymph node dissection to reach the target minimum of 12 resulted in upstaging three patients to stage III. The estimated cost to accurately stage these three patients is nearly $5168. One of these three cases was a pT4b tumor and was to get chemotherapy irrespective of pN stage. The other 2 cases were pT3 but with high risk features which according to NCCN guidelines would be considered for neoadjuvant therapy regardless. So, at least in our experience, the time, effort, and expense of achieving a target number of 12 lymph nodes likely had no effect on patient management. Clearly, there are a number of factors involved in the accurate assessment of lymph node involvement in patients with colorectal cancer. Our experience reflects that of a teaching hospital in which residents are involved in lymph node dissection in the operating theater and at the grossing bench. We recognize that setting a target number or goal is a helpful guideline, but we believe that good judgment and common sense must be exercised in determining the number of lymph nodes required to achieve accurate staging. However, we question the use of excessive resources in attempting to reach a relatively arbitrary target number that, at least in our experience, probably had little or no effect on patient management or outcome.

## Figures and Tables

**Table 1:** Mean number of lymph nodes retrieved in patients who received neoadjuvant therapy and who did not.

	Mean initial lymph nodes	*p* value	Mean total lymph nodes	*p* value
Neoadjuvant therapy	7.8 ± 5.1	0.61	14 ± 6.3	0.59
No therapy	8.7 ± 8.7		15.0 ± 9.0	

**Table 2:** Mean number of lymph nodes retrieved by residents compared to pathologist assistants.

	Mean initial lymph nodes	*p* value	Mean total lymph nodes	*p* value
Residents	8.4 ± 8.0	0.85	15.1 ± 8.3	0.29
Pathologist assistants	8.0 ± 6.0		13 ± 6.8	

**Table 3:** Cases in which additional lymph node search resulted in a change in pN.

Initial LN sections	Initial LN found	Initial LN positive	Additional LN sections	Additional LN found	Additional LN positive	Change in pN stage
13	8	2	31	9	1	ypN1a to ypN1b
10	16	1	4	1	1	pN1a to pN1b
7	11	0	4	6	2	pN0 to pN1b
10	9	0	107	18	0	pN0 to pN1c^∗^
4	4	0	13	11	3	pN0 to pN1b

^∗^One tumor deposit found in additional sections.

**Table 4:** Cases in which additional positive lymph nodes were found but pN stage remained the same.

Initial LN sections	Initial LN found	Initial LN positive	Additional LN sections	Additional LN found	Additional LN positive
10	14	5	7	13	1
12	9	6	4	9	2
6	6	4	16	10	1
9	11	6	11	3	1

**Table 5:** Summary of 55 cases in which second lymph node search helped to achieve a minimum of 12 lymph nodes.

Mean number of initial sections for lymph nodes	7.8 ± 5.8
Mean number of lymph nodes retrieved initially	7.1 ± 3.3 (1.3 ± 0.8/cassette)
Mean number of additional sections for lymph nodes	14.4 ± 15.8
Mean number of additional lymph nodes	9.0 ± 4.2 (1.0 ± 0.8/cassette)
Mean number of total lymph nodes	16.1 ± 3.2
